# ClpP protease modulates bacterial growth, stress response, and bacterial virulence in *Brucella abortus*

**DOI:** 10.1186/s13567-023-01200-x

**Published:** 2023-08-23

**Authors:** Dongjie Sun, Yufu Liu, Xiaowei Peng, Hao Dong, Hui Jiang, Xuezheng Fan, Yu Feng, Jiali Sun, Kun Han, Qiang Gao, Jianrui Niu, Jiabo Ding

**Affiliations:** 1 Institute of Animal Sciences, Chinese Academy of Agricultural Sciences, Beijing, China; 2 Zhaoqing Institute Biotechnology Co., Ltd., Zhaoqing, China; 3 Department of Inspection Technology Research, China Institute of Veterinary Drug Control, Beijing, China; 4 Institute for Laboratory Animal Resources, National Institutes for Food and Drug Control, Beijing, China; 5 Linyi University, Linyi, China

**Keywords:** *Brucella*, ClpP protease, virulence, RNA-seq, iTRAQ analysis

## Abstract

The process of intracellular proteolysis through ATP-dependent proteases is a biologically conserved phenomenon. The stress responses and bacterial virulence of various pathogenic bacteria are associated with the ATP-dependent Clp protease. In this study, a *Brucella abortus* 2308 strain, Δ*clpP*, was constructed to characterize the function of ClpP peptidase. The growth of the Δ*clpP* mutant strain was significantly impaired in the TSB medium. The results showed that the Δ*clpP* mutant was sensitive to acidic pH stress, oxidative stress, high temperature, detergents, high osmotic environment, and iron deficient environment. Additionally, the deletion of *clpP* significantly affected *Brucella* virulence in macrophage and mouse infection models. Integrated transcriptomic and proteomic analyses of the Δ*clpP* strain showed that 1965 genes were significantly affected at the mRNA and/or protein levels. The RNA-seq analysis indicated that the Δ*clpP* strain exhibited distinct gene expression patterns related to energy production and conversion, cell wall/membrane/envelope biogenesis, carbohydrate transport, and metabolism. The iTRAQ analysis revealed that the differentially expressed proteins primarily participated in amino acid transport and metabolism, energy production and conversion, and secondary metabolites biosynthesis, transport and catabolism. This study provided insights into the preliminary molecular mechanism between Clp protease to bacterial growth, stress response, and bacterial virulence in *Brucella* strains.

**Supplementary Information:**

The online version contains supplementary material available at 10.1186/s13567-023-01200-x.

## Introduction

Brucellosis is one of the most serious zoonotic diseases worldwide, caused by *Brucella* spp. In animals, this pathogen potentially causes abortion and male infertility. However, it exhibits different clinical symptoms in humans, such as Malta fever, night sweats, anorexia, polyarthritis, meningitis, and pneumonia [1].

In bacteria, the Clp protease is one of the key components of the proteolytic machinery, which consists of two main components: a ring-shaped proteolytic core complex composed of the Ser-type peptidase ClpP and a ring-shaped chaperone complex comprising the ATP-dependent chaperones ClpX and ClpA. Both ClpX and ClpA are members of the AAA + superfamily [2].

In many studies, ClpP and its chaperones have been shown to regulate several biological pathways, including protein homeostasis, cellular differentiation, stress response, and virulence. The mutation of ClpP or Clp-ATPases in *Escherichia coli* may result in a mild phenotype since both the Clp and Lon proteases are involved in protein homeostasis [3, 4]. Loss-of-function of ClpP in *Bacillus subtilis* inhibits motility, sporulation, and genetic competence. The *Bacillus subtilis clpP* mutant strain also shows reduced stationary phase survival and heat tolerance [5]. In *Staphylococcus aureus*, the Clp protease influences key biological processes such as the expression of virulence-associated genes, survival in the stationary phase, cell wall metabolism, and cell division [6]. ClpX and ClpP are essential for cell development, cell-cycle progression, chemotaxis, and bacterial replication in *Caulobacter crescentus* [7–10]. Several ClpXP substrates involved in cell-cycle progression have also been identified [11].

According to a previous study, *Brucella suis* exhibited induction of the protease protein ClpB under high-temperature conditions, despite not being the ATPase partner of ClpP protease. The *clpB* mutant displayed sensitivity to high temperature, ethanol stress, and acidic pH [12]. Furthermore, the ATP-dependent chaperone, ClpA, was involved in the degradation of the green fluorescent protein at 42 ℃. Furthermore, a double deletion of the *clpA* and *clpB* genes increased the sensitivity to oxidative stress [12]. Another study found that ClpA was involved in normal growth and response to heat stress in *B. suis*. Moreover, deletion of the *clpA* gene had little effect on the survival rate of cellular infection models [13]. However, the function of peptidase ClpP, one of the core components of Clp protease, is still unknown in *Brucella* spp.

In this study, a *clpP* mutant strain was constructed, and its survival rate at various stress conditions was studied. The virulence of this mutant strain was also evaluated in vitro and in vivo. Additionally, the integrated transcriptomic and proteomic analyses were performed to reveal the associated molecular mechanism of increased sensitivity to various stress conditions and virulence defects in the *clpP* mutant strain.

## Materials and methods

### Bacterial strains and culture conditions

*Escherichia coli* strains were cultured in Luria–Bertani broth at 37 ℃ with continuous shaking or on Luria–Bertani agar at 37 ℃. The *Brucella* strains were routinely grown in tryptic soy broth (TSB, BD company) or on tryptic soy agar medium and incubated at 37 ℃ with 5% CO_2_. *Brucella* strains (initial density of 1 × 10^6^ CFU/mL) were grown in TSB at 37 ℃ with continuous shaking for the growth curve assay. The OD_600_ of *B. abortus* 2308, Δ*clpP*, and C*clpP* (the complemented strain of Δ*clpP*) were measured at different intervals.

### Mice

Female 4–6 weeks old BALB/c mice (Beijing Vital River Laboratory Animal Technology Co., Ltd) were housed in cages with an air purification system, water, and food ad libitum.

### Construction of ΔclpP deletion mutant and genetic complementation

The *clpP* mutant strain was constructed according to the previously published protocol [14]. The resulting plasmid was termed plasmid pBluescript-*clpP*::Km.The Δ*clpP* mutant strain was created in *B. abortus* via an allelic exchange, following electroporation of the pBluescript-*clpP*::Km plasmid into the WT(wild type) strain 2308 and plated on TSB containing 50 μg/mL of kanamycin. Finally, ampicillin-sensitive and kanamycin-resistant colonies were verified by PCR to ensure that the gene of interest had been deleted while the kanamycin cassette was retained.

To construct the complemented strain (C*clpP*), the *clpP* fragment, including the promoter sequence (104 bp of the intergenic region upstream of the *clpP* start codon), was amplified using the primer pair C*clpP*-F/C*clpP*-R. The PCR products were then ligated to the pBBR1-MCS1 plasmid, and the final construct, named pBBR-*clpP*, was electroporated into the *clpP* deletion mutant strain. The cells were then plated onto TSA containing chlorampenicol (30 μg/mL) to isolate the complemented strain. The *B. abortus clpP* mutant and *clpP* complemented strains were verified by PCR, sequencing, and Western blotting. All primers used in this study are listed in Additional file 4.

### RNA isolation

For RNA isolation, *B. abortus* 2308 and the *clpP* mutant were grown in TSB at 37 ℃ until the stationary phase (about 2–5 × 10^9^ CFU/mL). Total RNA was isolated using TRIzol according to the manufacturer’s instructions. Residual DNA in the samples was removed by DNase I. RNA concentration and purity was determined using ND 1000 spectrophotometer (Thermo Scientific, Wilmington, USA).

### RNA-sequencing

The sequencing library of each RNA sample was prepared using the NEBNext Ultra Directional RNA Library Prep Kit for Illumina as per the manufacturer’s protocol. Briefly, RNA fragments were reverse-transcribed, amplified to double-stranded cDNA, and then ligated with an adaptor. The amplified cDNA was purified using a magnetic bead-based technique, and the molar concentration of each cDNA library was determined. The HiSeq 4000 platform was used to perform the transcriptome sequencing. Sequencing and subsequent bioinformatics analysis were completed at the Novel Bioinformatics Co., Ltd, Shanghai, China.

### Quantitative RT-PCR

Quantitative RT-PCR was performed as previously described [15]. The cDNA was generated from total RNA using a random hexamer primer and the PrimeScript^™^ II 1st Strand cDNA Synthesis Kit (TaKaRa, Dalian, Japan), according to the manufacturer’s instructions. Samples were run in triplicate and amplified in a 20 μL reaction containing 10 μL 2 × SYBR^®^ Premix Ex TaqTMII (TaKaRa, Dalian, China), 100 nM forward and reverse primers, and 1 μL appropriately diluted cDNA sample. 16S rRNA, which is constantly transcribed in bacteria, was selected as the housekeeping gene. qPCR was performed in triplicate for each gene, and relative transcription levels were determined using the 2^−∆∆Ct^ method [16]. Primers used for RT-qPCR are listed in Additional file 4.

### Crystal violet staining

*Brucella* might randomly lose the O-antigen of LPS during mutant construction, which was an important interference factor for assessing *Brucella* virulence [17, 18]. Therefore, to avoid this possibility, the crystal violet staining of the Δ*clpP* mutant strain was also performed.

For crystal violet staining, bacterial colonies on a plate were stained with 2 mL of 0.05% crystal violet for 15 s. The rough phenotype colonies take up the dye, whereas the smooth colonies do not [19].

### Cloning, protein expression, and purification

The coding region of the *clpP* gene (*bab1_1132*) was amplified using the primers r*ClpP*-For and r*ClpP*-Rev (Additional file 4). The amplified DNA fragment was double-digested with *Sac*I and *Hind*III restriction enzymes and ligated into double-digested plasmid pCold-SUMO. The resultant plasmid, pr*ClpP*, was transformed into *E. coli* strain BL21, and the strain harboring this plasmid was grown to an optical density of approximately 0.6 at 600 nm (OD_600_) before inducing recombinant gene expression by adding 0.1 mM IPTG. After 24 h of incubation at 16 ℃, the cells were collected by centrifugation (8000 × *g* for 10 min at 4 ℃) and lysed by treatment with 0.4 mg/mL lysozyme (TaKaRa, Dalian, China) and ultrasonic crushing. The supernatant from the lysed cell suspension was collected by centrifugation (12 000 × *g* for 20 min at 4 ℃) and passed through an affinity column packed with Ni Sepharose.

### Western blot analysis

*Brucella* strains from a single clone were grown in TSB medium for 72 h at 37 ℃. Then, 1 mL culture samples of each *Brucella* strain were centrifuged, suspended in 200 μL Laemmli sample buffer (Sigma), and boiled for 10 min. A 20 μL of each bacteria cell lysate was electrophoresed on 12% SDS–polyacrylamide gels. Resulting proteins were then transferred to polyvinylidene fluoride membranes (PVDF, Millipore) and detected using mouse anti-ClpP (dilution 1:500) or anti-OMP19 (dilution 1:500) polyclonal antibodies (which were generated in this study through immunization of BALB/c mice with recombinant ClpP or OMP19 proteins). Subsequently, peroxidase-conjugated secondary antibodies anti-mouse (dilution 1: 6000) (KPL) were used to develop ECL Plus Western Blotting Detection System (Amersham) membranes. Chemiluminiscence was detected by autoradiography.

### Stress assays

The susceptibility of *Brucella* strains under different stress conditions was assessed by the modified stress assays, as previously reported [20].

In the acidic stress assay, *Brucella* strains (with an initial density of 1 × 10^7^ CFU/mL) were cultivated for 2 h at 37 ℃ in acid peptone water (1 g/L Tryptone, 5 g/L NaCl, pH = 6.5, 5.5, or 4.5) with continuous shaking. Afterward, the concentration of bacteria was measured by plate count, and the survival rate (%) was calculated as the percentage of bacteria that survived in comparison to the TSB control.

The *Brucella* strains were treated with the same volume of H_2_O_2_ (freshly prepared in PBS) at final concentrations of 5, 2.5, and 1 mM in the oxidation stress assay. The concentration of bacteria was measured by plate count after 1 h of treatment at 37 ℃ without shaking, and the survival rate (%) was calculated as surviving bacteria relative to the PBS control.

To detect the resistance of *Brucella* strains to 0.01% SDS, 5 µL gradient dilution of bacterial cultures (1 × 10^9^ CFU/mL) were cultivated in TSA mediate in the presence and absence of 0.01% SDS for 72 h at 37 ℃ with 5% CO_2_.

To determine the sensitivity of *clpP* mutant to heat shock stress, 5 µL gradient dilution of bacterial cultures (1 × 10^9^ CFU/mL) were cultured on TSA medium at 42 or 37 ℃ with 5% CO_2_ for 72 h.

To determine the effect of *clpP* gene deletion on iron utilization, 5 µL gradient dilution of bacterial cultures (1 × 10^9^ CFU/mL) was cultivated in TSA medium in the presence and absence of 4 mM iron chelator, 2′2 dipyridyl for 72 h at 37 ℃ with 5% CO_2_.

To detect the effect of *clpP* gene deletion on hypertonic conditions, 5 µL gradient dilution of bacterial cultures (1 × 10^9^ CFU/mL) was cultivated in TSA medium in the presence and absence of 200 mM sodium chloride (NaCl) for 72 h at 37 ℃ with 5% CO_2_.

### Cellular infections

The in vitro intracellular viability of *B. abortus* 2308 and its derived strains was investigated using RAW264.7 cells. Cells were cultured in 24-well plates (Corning, NY, USA) and infected with *Brucella* strains at a multiplicity of infection (MOI) of 100, as previously described [21]. After 1 h of incubation, the cells were washed thrice with PBS and cultured in a medium containing gentamicin (50 μg/mL) to remove extracellular bacteria. Cells were washed three times with PBS again and lysed with 500 μL 0.1% (v/v) Triton X-100 water solution at 1, 12, 24, 48, and 72 h post-infection (hpi), and the number of bacteria surviving cells was determined by plating on TSA. The assays were run in triplicate and repeated at least twice.

### Mouse infections

To identify the virulence of *B. abortus clpP* mutant in the BALB/c mouse model, 40 female BALB/c mice aged 4–6 weeks were randomly divided into four groups. In group one, mice were intraperitoneally inoculated with 100 µL (1 × 10^5^ CFU) of *clpP* deficient mutant; In group two, mice were intraperitoneally inoculated with 100 µL (1 × 10^5^ CFU) of the parental strain *B. abortus* 2308; In group three, mice were intraperitoneally inoculated with 100 µL (1 × 10^5^ CFU) of the complemented strain C*clpP*; In group four, mice were intraperitoneally inoculated with PBS serving as the negative control. Mice were euthanized by asphyxiation (*n* = 5 per group) at 7 and 28 days post-infection (dpi). Their spleens were removed, weighed, and homogenized in 1 mL PBS. Bacterial colonies were enumerated by TSA plate count, and CFUs per spleen were calculated.

### Histopathology

Tissues from mice were examined to determine the association between the degree of pathology and *Brucella* strains. The animals were euthanized by CO_2_ asphyxiation, and their spleens, livers, and kidneys were harvested, fixed in 4% buffered formalin, embedded in paraffin, and stained with hematoxylin and eosin. The histological changes in the groups were compared. Sections from spleen and liver were graded in a blinded fashion (LS) on a scale of 0–4 for inflammation type and severity, as previously described [22].

### Isobaric tags for relative and absolute quantization (iTRAQ) analysis of proteins

The iTRAQ technology was carried out with the help of Gene Create Biolabs Inc (Wuhan, China). For this purpose, total proteins were isolated from each biological sample. The protein concentration was measured using the Bradford method. Following digestion with Trypsin Gold (Promega, Madison, WI, USA), the peptides were dried, reconstituted in 0.5 M triethylammonium bicarbonate (TEAB) buffer (Applied Biosystems, Milan, Italy), and then processed with 8-plex iTRAQ reagent (Applied Biosystems), according to the manufacturer’s protocol.

The mixed peptides were fractionated using the Ultimate 3000 HPLC system (Thermo DINOEX, USA). Mass spectrometry data were generated using the TripleTOF 5600 + liquid mass spectrometry system (SCIEX, United States) coupled with the Eksigent nanoLC system (SCIEX, United States). TripleTOF 5600plus liquid chromatography and mass spectrometry system (SCIEX) was used for mass spectrometry data acquisition.

The original MS/MS file data were analyzed by ProteinPilot Software v4.5, with the unused score ≥ 1.3 (corresponding to proteins identified with ≥ 95% confidence). An automatic decoy database search strategy was employed to estimate the false discovery rate (FDR) using the PSPEP (Proteomics System Performance Evaluation Pipeline Software, integrated with the ProteinPilot Software). Fold changes (FCs) were calculated as the average comparison pairs among biological replicates to determine differentially expressed proteins (DEPs). Proteins with an FC greater than 2 (upregulate ≥ 2.00 and down-regulate ≤ 0.50) and a Q-value less than 0.05 were considered significantly differentially expressed. The identified proteins were functionally annotated using the GO, COG, and KEGG databases. DEPs were performed GO and KEGG pathway enrichment analysis (*P*-value < 0.05).

### Statistical analyses

Basic statistical analyses were performed using SPSS 16.0 (SPSS Inc., Chicago, IL, USA)**.** An unpaired Student’s *t*-test was performed at each time point in growth curve assay, bacterial virulence assay of the *clpP* deficient strain in both cellular and mouse infection models. The Analysis of Variance (ANOVA) method was used for data analyses in the stress analysis. The data are presented as mean ± SD, and *p* values less than 0.05 were considered statistically significant.

## Results

### Construction of the ΔclpP mutant strain

A 478 bp fragment of the *BAB1_1132* coding gene sequence was deleted (Additional file 1). Western blotting was also performed, and appropriate size bands of the ClpP protein were only observed in the WT and C*clpP* strains, with no band observed in the *clpP* deficient strain (Additional file 1). Additionally, the crystal violet staining result indicated the LPS integrity was not affected in the *clpP* mutant strain (Additional file 2).

### Deletion of ClpP reduced the growth rate of the Brucella abortus 2308

**Figure 1:**
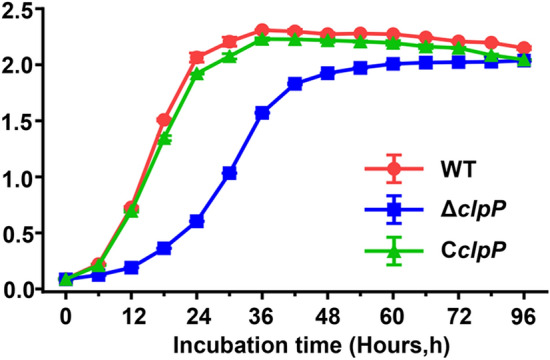
**Deletion of ClpP in** ***Brucella abortus*** **2308 resulted in a growth deficiency.** *Brucella* strains (initial density of 1 × 10^6^ CFU/mL) were grown in TSB at 37 ℃ with continuous shaking for 96 h. At different time points, OD_600_ of *B. abortus* 2308, Δ*clpP* and C*clpP* were measured.

We assessed the growth curves of WT, Δ*clpP*, and C*clpP* in TSB at 37 ℃ with shaking at 200 rpm for 96 h to characterize the role of *clpP* in *B. abortus* growth. The deletion of the *clpP* gene resulted in a reduced growth rate compared to WT and C*clpP*. The growth rate of the *clpP* mutant was significantly slower than WT and C*clpP* strains at the logarithmic phase (*p* < 0.001) (Figure 1), and it reached a stationary phase after about 60 h. However, in the TSB medium, it achieved a comparable stationary phase as the WT and C*clpP* strains.

### ClpP is required for survival under various stress conditions

**Figure 2:**
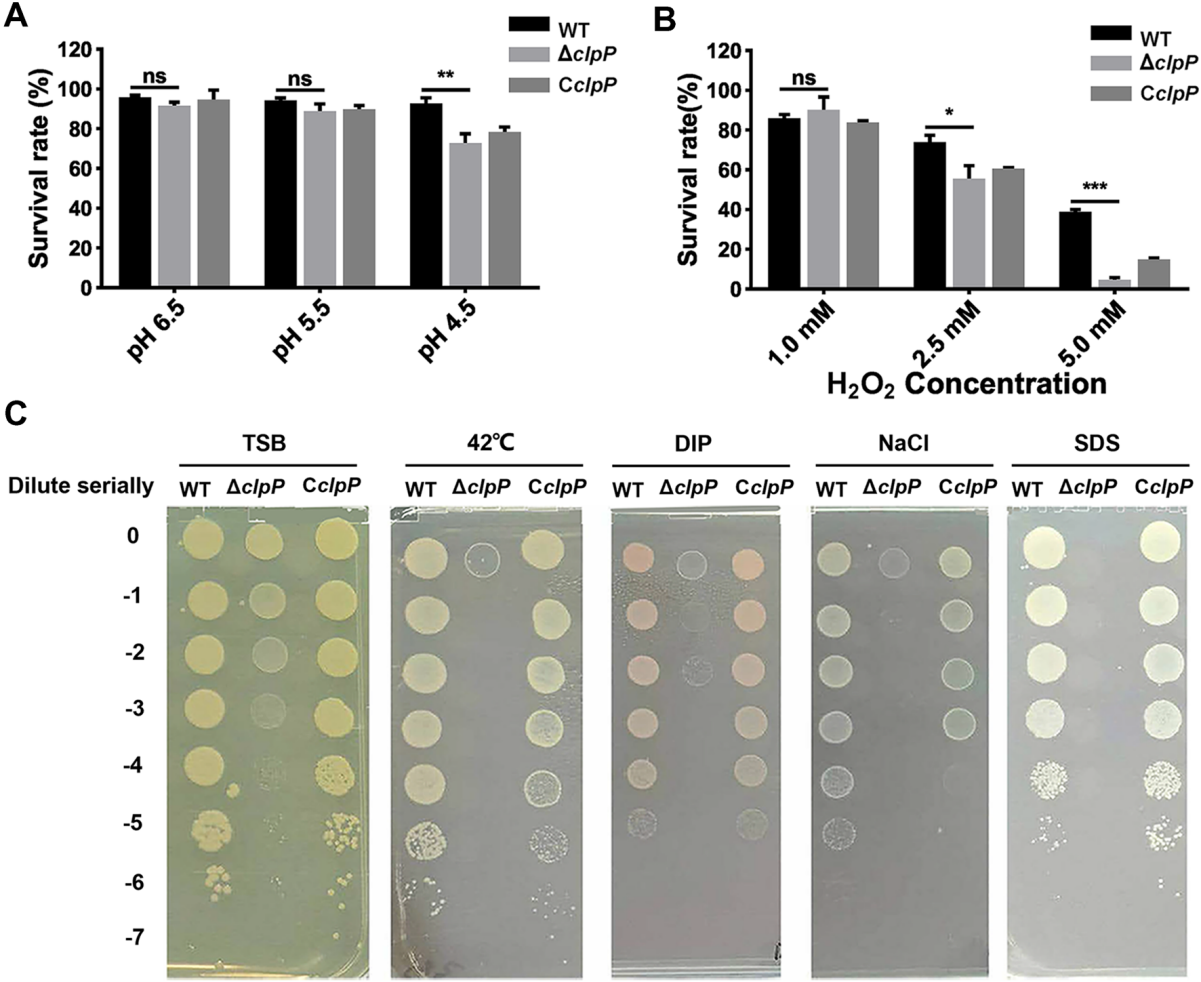
**Growth behavior of WT, Δ*clpP*** **and C*clpP*** **strains under various stress conditions.** **A** *Brucella* strains were cultivated in acid peptone water at 37 ℃ without shaking for 2 h. Viable counts were taken 2 h after the challenge. Values represent the mean from three independent experiments performed in duplicate. **B** 100 μL of the bacterial suspension was mixed with the same volume of H_2_O_2_ at final concentrations of 5 mM, 2.5 mM, and 1 mM. After 1 h of treatment at 37 ℃ without shaking, the concentration of bacteria was measured by plate count, and the survival rate (%) calculated as surviving bacteria relative to the PBS control. Values represent the mean from three independent experiments performed in duplicate. **C** 5 µL gradient dilution of bacterial cultures were, respectively, cultured on TSA medium at 42 ℃ for 72 h, TSA medium containing 4 mM iron chelator, 200 mM NaCl or 0.01% SDS at 37 ℃ for 72 h. These experiments were independently repeated thrice.

The survival ratios of the three strains (WT, Δ*clpP*, and *CclpP*) were comparable at pH 6.5 and 5.5 when exposed to acidic conditions. However, the survival ratio of Δ*clpP* was significantly lower than that of WT when subjected to pH 4.5 peptone water (*p* < 0.01) (Figure 2A).The survival ratio of Δ*clpP* was significantly reduced in comparison to WT when exposed to PBS containing 2.5 (*p* < 0.05) and 5 mM (*p* < 0.001) H_2_O_2_ (Figure 2B).To assess the significance of ClpP in heat stress tolerance, *Brucella* strains were cultured in TSA medium at 42 ℃ for 72 h. The results showed that the *clpP* mutant strain was more sensitive to heat stress than the WT and C*clpP* strains (Figure 2C). Additionally, when the *clpP* mutant strain was cultured on TSA medium supplemented with 4 mM iron chelator, 200 mM NaCl, or 0.01% SDS, the survival ratio of the mutant strain was also significantly reduced than that of WT and C*clpP* strains (Figure 2C).

### ClpP affected bacterial virulence in vivo and in vitro

**Figure 3:**
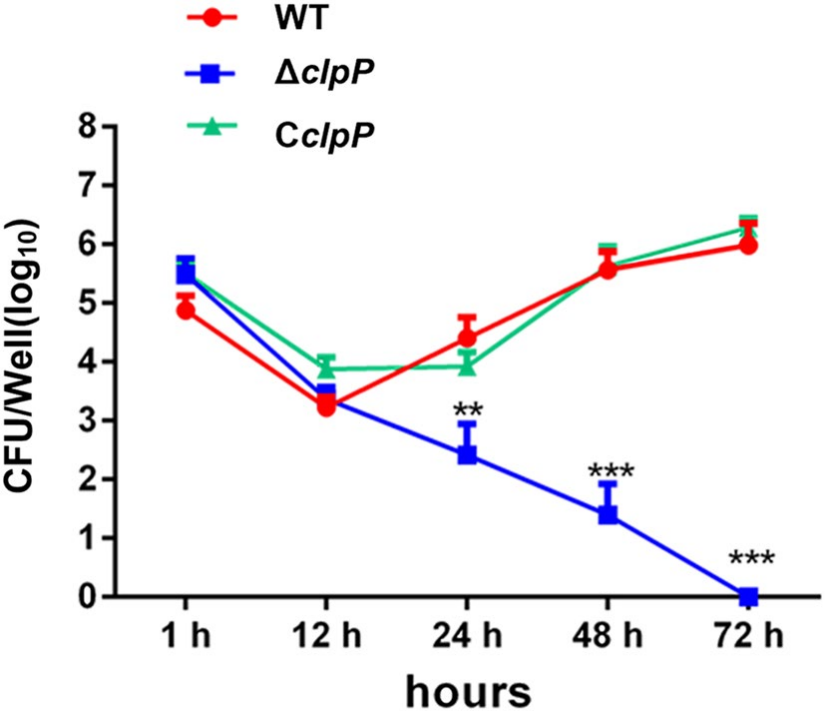
**The ClpP protease was involved in** ***Brucella*** **virulence in macrophage infection models.** Multiplication of *B. abortus* 2308, Δ*clpP* and C*clpP* strains in RAW264.7 macrophages over 72 h. Values represent the means of three experiments performed in duplicate, and error bars indicate the SD.

The multiplication ability of Δ*clpP* in RAW264.7 cells was tested to determine the role of ClpP in virulence. As shown in Figure 3, the intracellular bacterial load of the Δ*clpP* strain was significantly reduced after 24 (*p* < 0.01), 48 (*p* < 0.001), and 72 h (*p* < 0.001) in murine macrophages RAW264.7 compared to the parent strain control.

**Figure 4:**
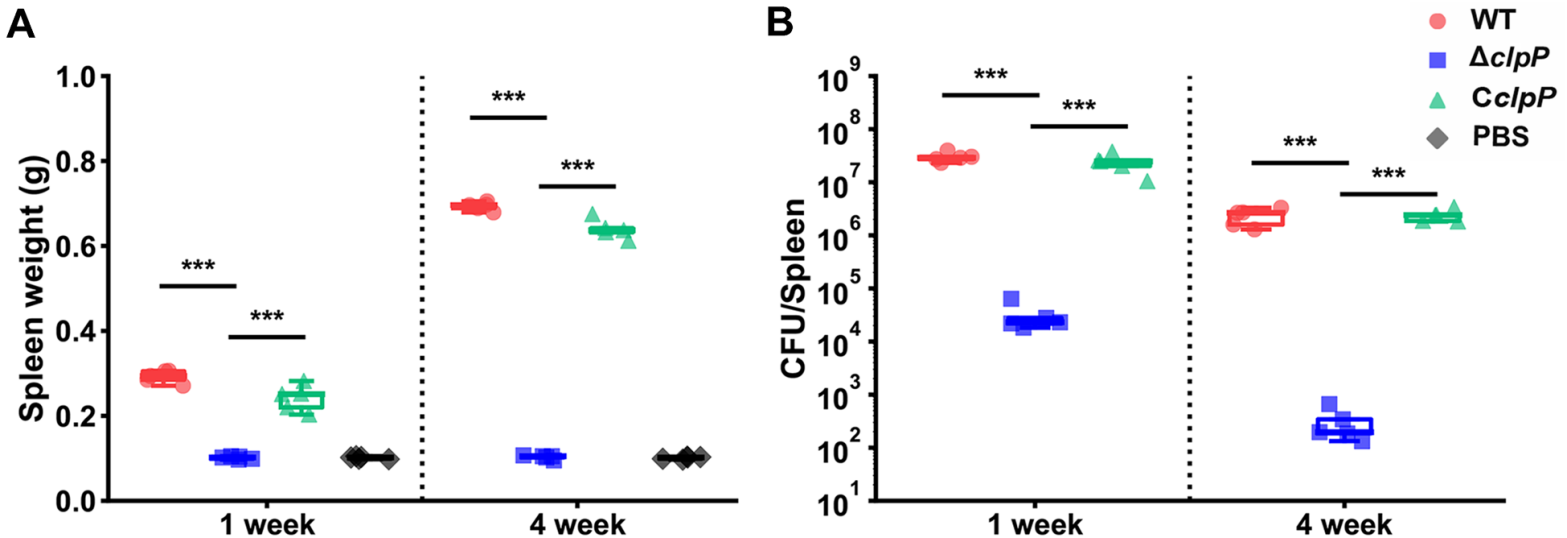
**The ClpP protease was involved in** ***Brucella*** **virulence in mice infection models.** **A** The spleen weight of Δ*clpP*, C*clpP*, and WT infected mice at 1 and 4 weeks post-infection. **B** The splenic CFUs of Δ*clpP*, C*clpP*, and WT infected mice at 1 and 4 weeks post-infection.

To further characterize the role of ClpP in virulence, the survival rates of the Δ*clpP,* C*clpP*, and WT strains were assessed in a mouse infection model at one and four weeks. Figure 4A demonstrates that the spleen weight of the mice with *clpP* deletion mutant infected mice was significantly lower compared to the WT and C*clpP* strains at one and four weeks post-infection (*p* < 0.001). Moreover, the Δ*clpP* strain recovered from the spleens of infected mice was significantly reduced at one and 4 weeks post-infection (*p* < 0.001) (Figure 4B).

### Evaluation of histological changes in mice infected with ΔclpP

**Figure 5:**
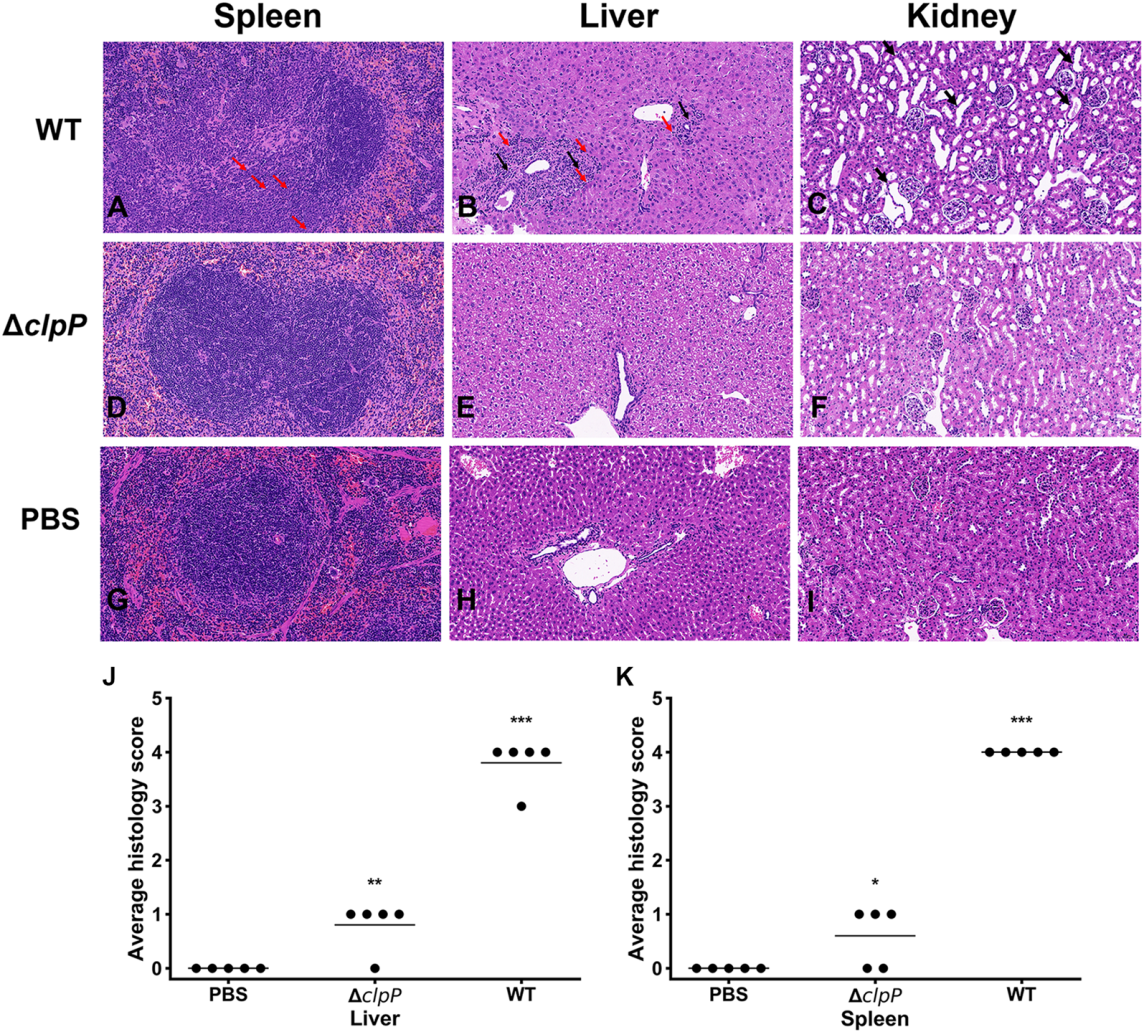
**Histological changes of organs associated with infection.** At 4 weeks post infection, tissues of mice infected with WT or Δ*clpP* strain were collected. The histology of animals infected with WT (**A**, **B**, and **C**) were compared to animals infected with Δ*clpP* (**D**, **E**, and **F**). Naive mice are depicted (**G**, **H**, and **I**) for comparison. Hematoxylin and eosin staining was used (× 20 magnification). Sections from liver and spleen were scored for severity of histiocytic inflammation from 1 to 4 at 4 weeks post-infection, and mean scores were compared using unpaired Student’s *t*-test (**J** and **K**). One asterisks, *p* < 0.05, two asterisks, *p* < 0.01, and three asterisks, *p* < 0.001.

The degree of inflammation after four weeks of infection elicited by different virulent strains of *Brucella* was determined by histologically analyzing the spleen, liver, and kidney tissues of BALB/c mice infected with WT (Figures 5A–C), Δ*clpP* (Figure 5D–F), and naive PBS controls (Figures 5G–I). Histologically, the number of spleen nodules and splenomegaly lymphocytes reduced, while the number of macrophages and neutrophils (red arrows) increased (Figure 5A). No significant inflammatory response was observed in the spleen of the mice infected with the *clpP* mutant (Figure 5D) compared to the PBS control group (Figure 5G). Connective tissue hyperplasia (black arrows) and inflammatory cell infiltration (red arrows) was observed in the livers of mice infected with WT (Figure 5B); however, it was absent in mice infected with the *clpP* mutant (Figure 5E) or the negative control (Figure 5H). The kidneys of mice infected with WT showed a clear expansion of distal convoluted renal tubule (black arrows) (Figure 5C) but were minimal in the mice infected by Δ*clpP* strain (Figure 5F). In addition, a grading system was used to characterize histopathologic changes in the spleen and liver. Calculation of histologic scores for each organ revealed that a significant increase (*p* < 0.001) in lesion severity was observed in the WT infection group, and the histopathologic scores of Δ*clpP* infection group were lower than the WT infection group. Nevertheless, the histopathologic scores of Δ*clpP* infection group remained significantly higher than uninfected mice group in liver (*p* < 0.01) and spleen (*p* < 0.05) (Figures 5J, K).

### Transcriptomic analysis

**Figure 6:**
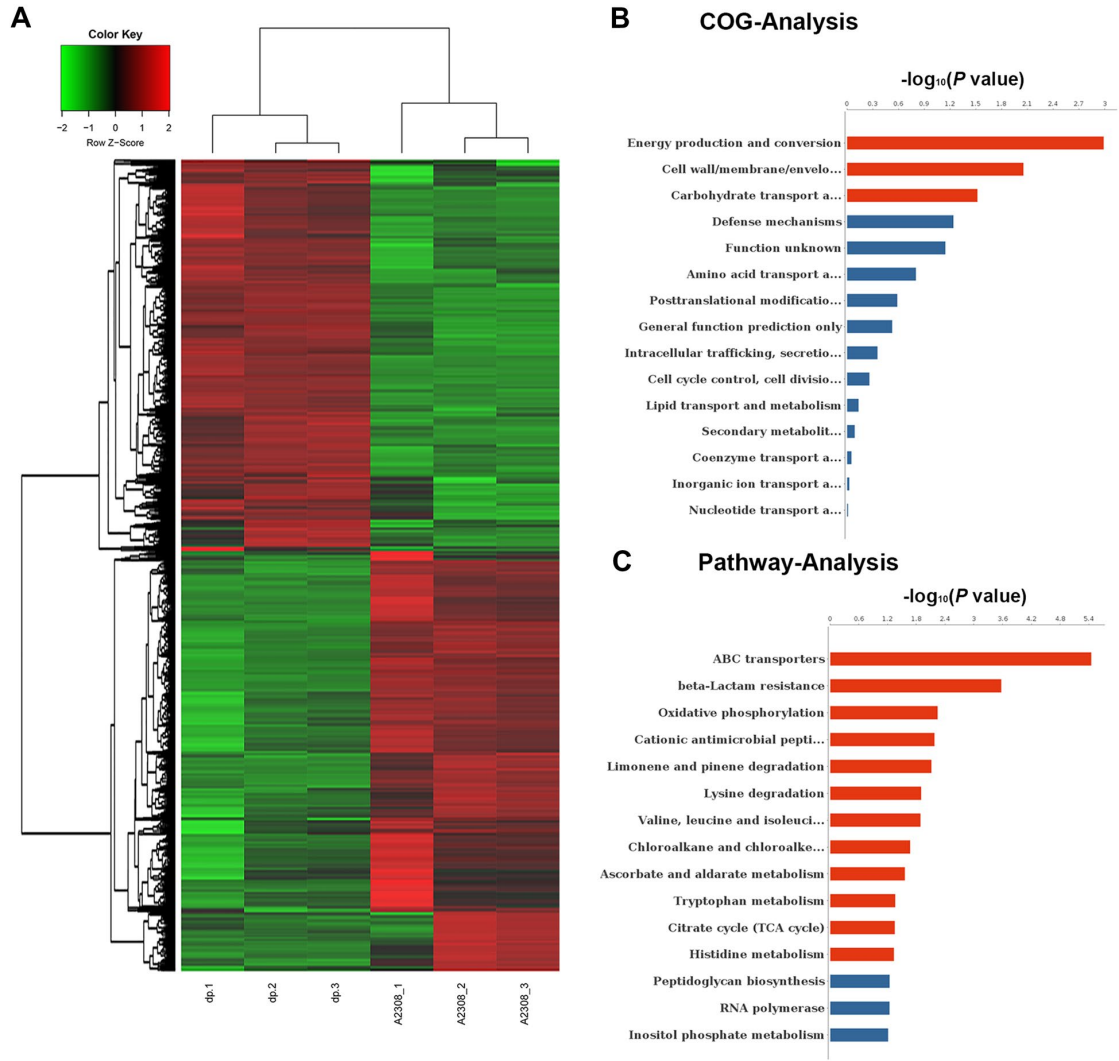
**Transcriptomic analysis for differentially expressed genes.** **A** Heat map of the two-way hierarchical clustering. **B** COG analysis of the DEGs between WT and Δ*clpP*. **C** KEEG analysis of the DEGs between WT and Δ*clpP*.

The spectrum of *Brucella* genes affected by the ClpP protein and the genetic basis for the distinct phenotypic properties exhibited by the Δ*clpP* strain was investigated by using RNA-seq analysis of the total RNA isolated from the WT and the Δ*clpP* strains grown in TSB until early stationary phase (about 2–5 × 10^9^ CFU/mL). A total of 1779 genes exhibited differential expression (Fold change > 2 and FDR < 0.05) in the *clpP* mutant (Additional file 5). The RNA-seq results were verified by RT-qPCR (Additional file 3). The *clpP* mutant strain showed upregulation of 858 genes and downregulation of 921 genes (Figure 6A). The COG analysis revealed that the differentially expressed genes were primarily involved in energy production and conversion, cell wall/membrane/envelope biogenesis, and carbohydrate transport and metabolism (Figure 6B). KEGG pathway analysis demonstrated that the genes which displayed significant differences were mainly enriched in various pathways such as ABC transporters, beta-Lactam resistance, oxidative phosphorylation, cationic antimicrobial peptide (CAMP) resistance, limonene and pinene degradation, lysine degradation, valine degradation, leucine, and isoleucine degradation, chloroalkane and chloroalkene degradation, ascorbate and aldarate metabolism, tryptophan metabolism, citrate cycle (TCA cycle), and histidine metabolism (Figure 6C). The RNA-seq results revealed that the top 10 upregulated genes were BAB2_0942, BAB2_0943, BAB2_0050, BAB2_0953, *hemN*-2, BAB2_0952, BAB1_1513, BAB1_0736, BAB1_0622, and BAB2_0929, while the top 10 down-regulated genes BAB2_0277, BAB1_1215, BAB1_0041, BAB2_0276, BAB2_0278, BAB1_1214, BAB1_0040, *rplI*, BAB1_1964, and *rpsN*.

### Proteomic analysis

**Figure 7:**
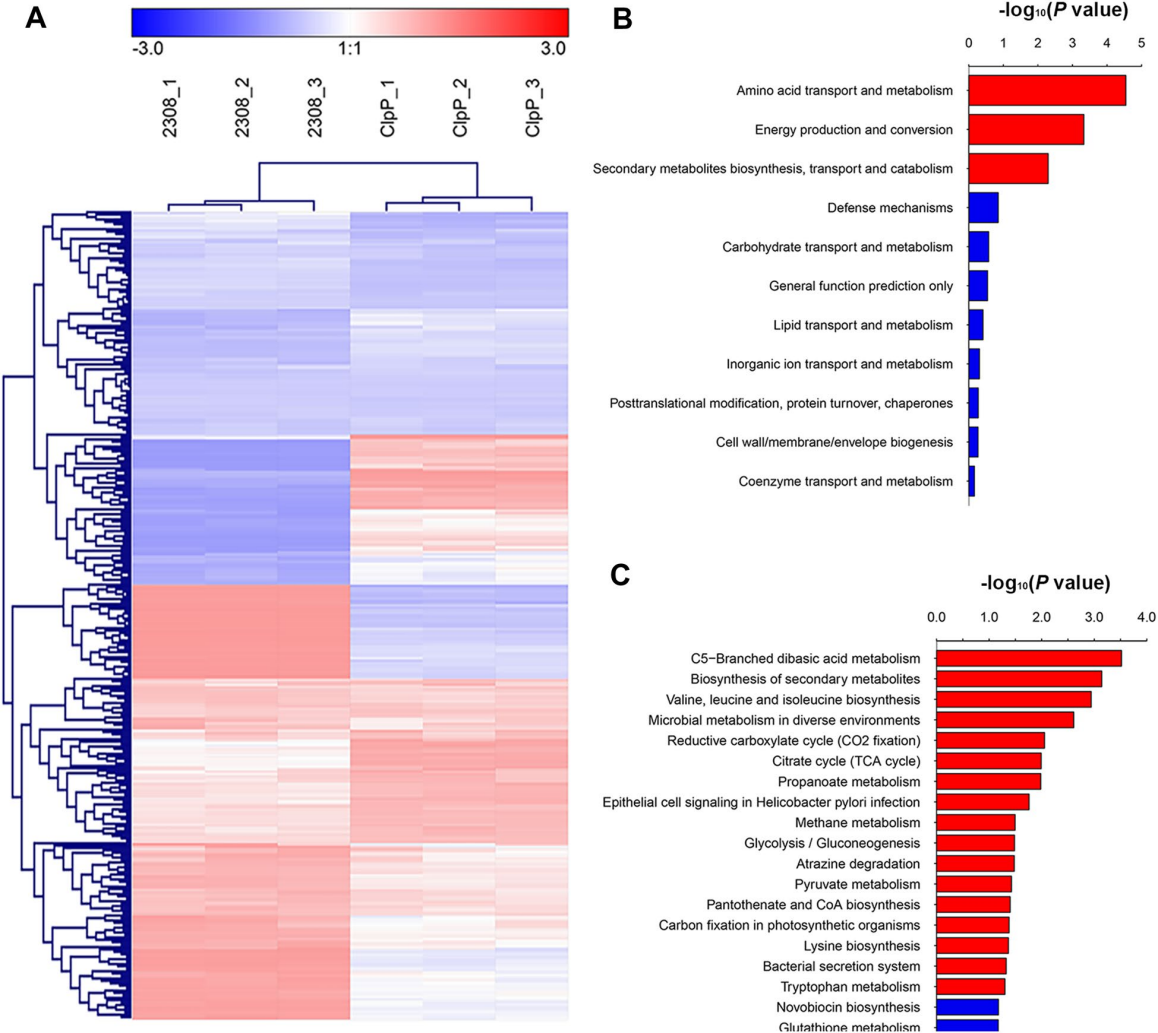
**Proteomic analysis for differentially expressed genes.** **A** Heat map of the two-way hierarchical clustering. **B** COG analysis of the DEPs between WT and Δ*clpP*. **C** KEEG analysis of the DEPs between WT and Δ*clpP*.

Given the prominent phenotypes induced by *clpP* deletion, the proteomes of WT and the Δ*clpP* strain were compared. A total of 2066 proteins were identified in both strains, and 337 significantly affected proteins were found to be differentially expressed in the Δ*clpP* strain (Additional file 6). Among differentially expressed proteins (DEPs), 182 were identified to be upregulated, while 155 were downregulated (Figure 7A). According to the results of COG analysis, the DEPs were mainly involved in amino acid transport and metabolism, energy production and conversion, and secondary metabolites biosynthesis, transport, and catabolism (Figure 7B). The KEGG pathway analysis demonstrated that the genes exhibited a significant difference were primarily enriched in various metabolic pathways, including C5-branched dibasic acid metabolism, biosynthesis of secondary metabolites, valine leucine, and isoleucine biosynthesis, microbial metabolism in diverse environments, and reductive carboxylate cycle (Figure 7C).

According to the proteomic analysis, the top 10 upregulated proteins were BAB2_0880, RibH2, BAB1_0239, BAB1_1744, UreG2, UbiG, BAB1_0241, SucC, BAB1_1964, and Bpt, while the top 10 down-regulated proteins were ClpS, BAB1_0115, BAB1_0018, BAB1_0296, BAB1_0875, BAB1_1435, BAB1_1357, BAB2_0019, BAB1_1214, and BAB2_0547.

### Integrated analysis revealed the global roles of ClpP in B. abortus 2308

According to the proteomic and transcriptomic profiles, *clpP* deletion extensively affected the physiology of WT at both the protein and mRNA levels. Therefore, we integrated transcriptomic and proteomic analyses to explore the comprehensive effects of *clpP* deletion on WT.

**Figure 8:**
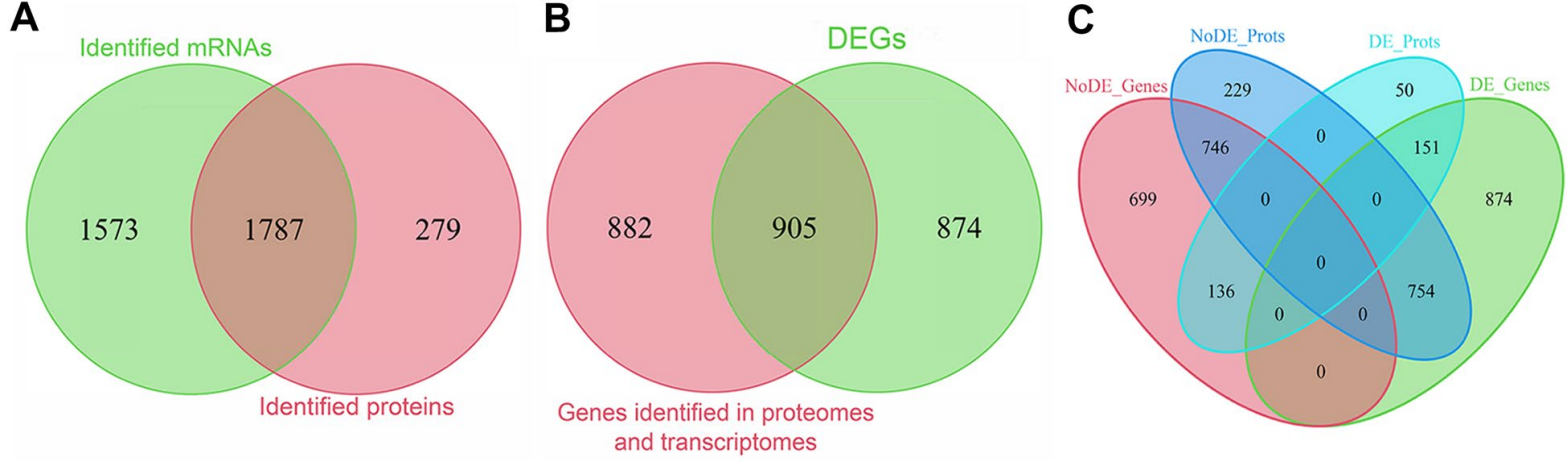
**Integrated proteomic and transcriptomic analyses.** **A** Venn diagram showing that the 1787 proteins identified in the proteomes can be assigned to the 3360 mRNAs identified in the transcriptomes. The green circle indicates all mRNAs identified in both Δ*clpP* and WT; the pink circle indicates all proteins identified in both Δ*clpP* and WT. **B** Venn diagram showing that 905 DEGs were included in the 1787 genes identified in both the proteomes and transcriptomes. The green circle indicates DEGs in Δ*clpP*, the pink circle indicates 1787 genes identified in both the proteomes and transcriptomes. **C** Venn diagram showing the effects of *clpP* deletion on WT strain at the mRNA and/or protein levels. The green ellipse indicates genes significantly affected by *clpP* deletion in the transcriptomic profiles; the turquoise ellipse indicates proteins significantly affected by *clpP* deletion in the proteomic profiles; the pink ellipse indicates genes not affected by *clpP* deletion in the transcriptomic profiles; the blue ellipse indicates proteins not affected by *clpP* deletion in the proteomic profiles.

The transcriptomic and proteomic profiles identified 3360 mRNAs and 2066 proteins, respectively, of which 1787 identified proteins were assigned to the identified mRNAs (Figure 8A and Additional file 7). Of all the differentially expressed genes (DEGs) identified in the Δ*clpP* strain, 905 were included in the 1787 genes identified in both the proteomes and transcriptomes (Figure 8B). As shown in Figure 8C, 1965 genes were significantly affected at the mRNA and/or protein levels in the Δ*clpP* strain. Among the 1965 genes, 136 were found to be significantly affected only at the protein level (NoDE_Genes and DE_Prots), 754 were significantly affected only at the mRNA level (DE_Genes and NoDE_Prots), and 151 were significantly affected at both the mRNA and protein levels (DE_Genes and DE_Prots) (Figure 8C).

Up to 71 NoDE_Genes and upregulated DE_Prots genes in the Δ*clpP* strain indicated that certain proteins might serve as substrates for direct degradation by Clp proteases. Consequently, the presence of 754 genes affected only at the mRNA level (DE_Genes and NoDE_Prots genes) suggested the possibility of a *clpP*-independent regulation pathway at the protein level to overcome the altered gene transcriptions and restore the corresponding proteins to a physiological level. A total of 86 DE_Genes and DE_Prots genes exhibited regulation in the same direction at both the mRNA and protein levels, suggesting the ClpP protein may have an impact only at the mRNA level of these genes. However, 65 of 151 genes affected at both mRNA and protein levels (DE_Genes and DE_Prots) showed opposite regulation trends at the mRNA and protein levels, suggesting the possibility of a predominant and ClpP-dependent protein regulation pathway that could potentially reverse the transcriptional alterations caused by the *clpP* gene mutation.

## Discussion

Bacteria are exposed to a variety of environmental stresses that can directly or indirectly affect the protein structure, activity, and homeostasis. Misfolded proteins could become aggregated in bacteria, resulting in toxicity to the bacteria. The damaged, misfolded, or aggregated proteins that cannot be repaired are rapidly degraded by the AAA + protease complexes [23]. The Clp protease system is one of the main components of the AAA + protease complexes, which is highly conserved within both bacterial domain and functionally organized in two separate compartments.

### The clpP gene is a non-essential gene in Brucella abortus

In a recent study, multidimensional transposon sequencing analysis revealed that the *clpP* gene is an essential gene of *Brucella* spp. However, some other published studies showed successful deletion of a few essential genes identified in this study in *brucella* strains, including *mucR*, *sodA*, *pgm*, and *wbkC* [24, 25]. Additionally, the presence of the putative SocAB toxin-antitoxin system was also considered, which renders the ClpXP system essential in *Brucella* strains [24, 25]. To date, no study has been published on the SocAB toxin-antitoxin system in *Brucella* strains. However, a study on the SocAB toxin-antitoxin system was performed in *Caulobacter crescentus*, in which ClpX and ClpP were essential for viability, unlike in most bacteria [26].

In this study, a *clpP* marked deletion mutant strain was successfully constructed for the first time and verified through sequencing and western blot analysis. In addition, in this mutant strain, the transcription of flanking genes of *clpP* was not affected. Accordingly, the *clpP* gene was considered to be non-essential for the survival of *Brucella* spp.

### ClpP-deletion impairs the virulence of Brucella abortus

In many pathogenic bacteria, ClpP peptidase is the core component of the Clp protease system and is involved in bacterial virulence. ClpP is essential in the intracellular growth of *Legionella pneumophila* in macrophages [27]. The *clpP* mutant of *Staphylococcus aureus* has been found to have attenuated intracellular replication in models of systemic and abscess infection [28–31]. Besides, this study also demonstrated that the ClpP protease played an important role in *Brucella* virulence.

Previous studies have examined the functions of various components of protease complexes, such as Lon, ClpA, and ClpB. The survival rate of the *lon* deletion strain reduced after a one-week infection of BALB/c mice [32, 33]. The ATP-dependent chaperone ClpA did not correlate with *Brucella* virulence, but its absence significantly increased the persistence of *B. suis* in a mice infection model [13]. Unlike these *Brucella* protease components, deletion of the ClpP protein significantly reduced *Brucella* virulence in both macrophage and mouse infection models. Moreover, the histopathology assay results indicated that infection caused by the *clpP* mutant strain does not cause severe inflammation in mice compared to that of the WT strains. Importantly, a correlation between proteolysis and *Brucella* virulence was confirmed by the results of our study for the first time.

Previous research indicated that the *virB* operon mediated BCVs evasion of lysosomal degradation [34]. The RNA-seq and qPCR results indicated that the transcriptional level of the *virB* operon was significantly downregulated in the *clpP* mutant strain (Additional file 3). Thus, the abnormal expression of the *virB* operon might be related to the inability of the mutant strain to escape host immunity in macrophages.

### ClpP globally affects the gene expression pattern

Previous studies have conducted proteomic and transcriptomic analyses to uncover the function of the ClpP protein. The results indicated that this protease could affect numerous genes and proteins across diverse pathways globally. In our study, a total of 1965 genes were found to be significantly affected at the mRNA and/or protein levels in the Δ*clpP* strain, and our results also supported the important roles of the ClpP protein. Additionally, the RNA-seq data showed an upregulation of several genes involved in protein homeostasis, including *dnaK*, *dnaJ*, *clpB*, and *groES*. These genes were also found to be upregulated in previous proteomic and transcriptomic analyses [35, 36]. According to our proteomic data, several proteases (Lon, Ftsh, and BAB2_0915) were also upregulated, which seemed to be a probable functional compensation.

### The sensitivity of the clpP mutant strain to various stress conditions is an important reason for its reduced virulence

As a facultative intracellular pathogen, *B. abortus* 2308 could encounter various formidable stress conditions during its interactions with its host cells. These stress conditions include exposure to reactive oxygen and nitrogen species, acidic pH, and nutritional deprivation. Iron is also an essential metal element required for the optimal growth of *Brucella* strains in vitro and for the bacterial virulence of *Brucella* strains in animal infection models [37]. To further study the underlying cause of decreased virulence of the *clpP* mutant strain, we comprehensively analyzed the stress response of the *clpP* mutant strain in different stress conditions. Our results showed that the *clpP* mutant strain was more sensitive to heat stress, iron deficiency, detergents, and hypertonic environment. Consequently, it was assumed that the attenuation of the *clpP* deficient strain was possibly due to its reduced tolerance in stress conditions.

The ECF sigma factor SigE1 (also known as RpoE1) may suppress flagellar synthesis and filament length in *B. melitensis* [38]*.* The *rpoE1* mutant strain of *B. abortus* was more sensitive to oxidative and acid stress [24]. Additionally, the *B. abortus* and *B. melitensis* mutants lacking the *rpoE1* gene were attenuated in the chronic phase of the BALB/c mice infection model [39, 40]. According to the proteomes data, the RpoE1 protein level was significantly reduced in the Δ*clpP* mutant strain. Considering the important role of RpoE1 protein in *Brucella* strains, its abnormal expression likely caused the reduced virulence of the mutant strain.

According to the results of the stress assay, the Δ*clpP* mutant was more sensitive to detergents (SDS) and hypertonic conditions. This was consistent for 51 differently expressed genes involved in cell wall/membrane/envelope biogenesis in the Δ*clpP* mutant strain. ClpP was not related to the bacterial lipopolysaccharide biosynthesis, as evidenced by the fact that the Δ*clpP* mutant was a smooth-type *Brucella* strain. In previous studies, numerous outer membrane-related genes (*omp10*, *omp19*, *bepC*, *bacA*, *ugpB*, and *cgs*) were found for the overall virulence of *Brucella* strains, but none of these genes were significantly differentially expressed at mRNA and protein levels in the Δ*clpP* mutant [39].

Recent research suggested that the ClpP protein in *Staphylococcus aureus* could mediate iron-acquiring systems [41], and the ClpP protease also affected the iron acquisition ability of *Actinobacillus pleuropneumoniae* [42]. Our study showed that the *clpP* mutant strain was more sensitive to iron deficiency stress in *Brucella abortus*. Surprisingly, the protein level expression of up to nine iron metabolism-related varied. Except for compensatory up-regulation of 2,3-DHBA/brucebactin transport Fiu (encoding by *BAB2_0233*), eight DEPs involved in biosynthesis of 2,3-DHBA (*BAB2_001*2, *BAB2_0013*, and *BAB2_0014*), Heme transport (*bhuA*), 2,3-DHBA/brucebactin transport (*BAB2_0564*), Fe^3+^ transport (*BAB2_0519* and *BAB2_0539*), and bacterioferritin (bfr) were all down-regulated in the *clpP* mutant. Additionally, the transcript levels of the other eight DEPs associated with iron metabolism were not significantly affected in our RNA-seq assay, except for the upregulated *bhuA* gene; indicating that these DEPs may be indirectly affected by ClpP protease at the processes of protein translation, stability, or degradation. In a previous study, it was demonstrated that the Heme transporter BhuA played an important role in the stationary-phase iron utilization in *B. abortus* 2308. Moreover, the *bhuA* mutant strain exhibited significant attenuation in the macrophage infection model and it was also unable to maintain a chronic infection in BALB/c mice [43]. Therefore, the differential expression of these iron metabolism-related DEPs in the mutant strain seemed to be the primary cause for the sensitivity to iron-deficient conditions and reduced virulence in vitro and in vivo.

ClpP protease has been proved to affect the virulence and infectivity of various pathogens, this protein is considered as a promising novel antibacterial target. Currently, targeting ClpP-ATPases interaction and ClpP inhibition are the most successful and advanced strategy [44]. In this study, the ClpP protein is also closely involved in virulence in *Brucella*, which indicates that this protease has potential applications in the development of therapeutic drugs for *brucellosis*.

In conclusion, the current study showed that ClpP was essential for bacterial normal growth, tolerance to various stress conditions, and *Brucella* virulence. Additionally, several genes involved in the phenotypic changes of the *clpP* mutant were identified using RNA-seq and iTRAQ assay. This study revealed the preliminarily molecular mechanism of ClpP protease, stress response, and *Brucella* virulence.

### Supplementary Information

**Additional file 1: Characterization of the mutant strain ∆clpP.** (A) Schematic of BAB1_1132 gene deletion. A 478-bp fragment was deleted from the BAB1_1132 coding sequence. (B) Westen blot assay of ClpP protein expression in different strain. Lane WT: protein sample of B.abortus 2308; Lane ∆clpP: protein sample of ∆clpP strain; Lane CclpP: protein sample of CclpP strain.

**Additional file 2: The crystal violet staining of Brucella strains.** The brucella strains (2308, ∆clpP, CclpP, and RM6/66) were stained with 0.05% crystal violet. RM6/66 was a rough strain, which picked up the crystal violet dye.

**Additional file 3: Transcriptional level of 36 selected genes by RNA-seq and RT-qPCR.**

**Additional file 4: Primers used in this study.**

**Additional file5: RNA-seq analysis results of WT and the ΔclpP strain.**

**Additional file 6:** **Proteomic Analysis results of WT and the ΔclpP strain.**

**Additional file 7: Integrated Analysis results of WT and the ΔclpP strain.**

## Data Availability

The transcriptional data set had been submitted to the GEO repository (NCBI) with access number GSE126915. The mass spectrometry proteomics data have been deposited to the ProteomeXchange Consortium via the PRIDE partner repository with the dataset identifier PXD034842.

## References

[1] Olsen SC, Palmer MV (2014). Advancement of knowledge of *Brucella* over the past 50 years. Vet Pathol.

[2] Sauer RT, Baker TA (2011). AAA+ proteases: ATP-fueled machines of protein destruction. Annu Rev Biochem.

[3] Wu WF, Zhou Y, Gottesman S (1999). Redundant in vivo proteolytic activities of *Escherichia coli* Lon and the ClpYQ (HslUV) protease. J Bacteriol.

[4] Smith CK, Baker TA, Sauer RT (1999). Lon and Clp family proteases and chaperones share homologous substrate-recognition domains. Proc Natl Acad Sci U S A.

[5] Msadek T, Dartois V, Kunst F, Herbaud ML, Denizot F, Rapoport G (1998). ClpP of *Bacillus subtilis* is required for competence development, motility, degradative enzyme synthesis, growth at high temperature and sporulation. Mol Microbiol.

[6] Frees D, Gerth U, Ingmer H (2014). Clp chaperones and proteases are central in stress survival, virulence and antibiotic resistance of *Staphylococcus aureus*. Int J Med Microbiol.

[7] Biondi EG, Reisinger SJ, Skerker JM, Arif M, Perchuk BS, Ryan KR, Laub MT (2006). Regulation of the bacterial cell cycle by an integrated genetic circuit. Nature.

[8] Iniesta AA, McGrath PT, Reisenauer A, McAdams HH, Shapiro L (2006). A phospho-signaling pathway controls the localization and activity of a protease complex critical for bacterial cell cycle progression. Proc Natl Acad Sci USA.

[9] Lesley JA, Shapiro L (2008). SpoT regulates DnaA stability and initiation of DNA replication in carbon-starved *Caulobacter crescentus*. J Bacteriol.

[10] Gorbatyuk B, Marczynski GT (2005). Regulated degradation of chromosome replication proteins DnaA and CtrA in *Caulobacter crescentus*. Mol Microbiol.

[11] Bhat NH, Vass RH, Stoddard PR, Shin DK, Chien P (2013). Identification of ClpP substrates in *Caulobacter crescentus* reveals a role for regulated proteolysis in bacterial development. Mol Microbiol.

[12] Ekaza E, Teyssier J, Ouahrani-Bettache S, Liautard JP, Köhler S (2001). Characterization of *Brucella suis* clpB and clpAB mutants and participation of the genes in stress responses. J Bacteriol.

[13] Ekaza E, Guilloteau L, Teyssier J, Liautard JP, Köhler S (2000). Functional analysis of the ClpATPase ClpA of *Brucella suis*, and persistence of a knockout mutant in BALB/c mice. Microbiology.

[14] Liu WJ, Dong H, Peng XW, Wu QM (2014). The Cyclic AMP-binding protein CbpB in *Brucella melitensis* and its role in cell envelope integrity, resistance to detergent and virulence. FEMS Microbiol Lett.

[15] Liu W, Dong H, Liu W, Gao X, Zhang C, Wu Q (2012). OtpR regulated the growth, cell morphology of *B. melitensis* and tolerance to β-lactam agents. Vet Microbiol.

[16] Livak KJ, Schmittgen TD (2001). Analysis of relative gene expression data using real-time quantitative PCR and the 2(-Delta Delta C(T)) method. Methods.

[17] Turse JE, Pei J, Ficht TA (2011). Lipopolysaccharide-deficient *Brucella* variants arise spontaneously during infection. Front Microbiol.

[18] Lapaque N, Moriyon I, Moreno E, Gorvel JP (2005). *Brucella lipopolysaccharide* acts as a virulence factor. Curr Opin Microbiol.

[19] White PG, Wilson JB (1951). Differentiation of smooth and nonsmooth colonies of *Brucellae*. J Bacteriol.

[20] Dong H, Peng X, Liu Y, Wu T, Wang X, De Y, Han T, Yuan L, Ding J, Wang C, Wu Q (2018). BASI74, a virulence-related sRNA in *Brucella abortus*. Front Microbiol.

[21] Bao Y, Tian M, Li P, Liu J, Ding C, Yu S (2017). Characterization of *Brucella abortus* mutant strain Δ22915, a potential vaccine candidate. Vet Res.

[22] Stranahan LW, Khalaf OH, Garcia-Gonzalez DG, Arenas-Gamboa AM (2019). Characterization of Brucella canis infection in mice. PLoS ONE.

[23] Mogk A, Huber D, Bukau B (2011). Integrating protein homeostasis strategies in prokaryotes. Cold Spring Harb Perspect Biol.

[24] Kim HS, Caswell CC, Foreman R, Roop RM, Crosson S (2013). The *Brucella abortus* general stress response system regulates chronic mammalian infection and is controlled by phosphorylation and proteolysis. J Biol Chem.

[25] Sternon JF, Godessart P, Gonçalves de Freitas R, Van der Henst M, Poncin K, Francis N, Willemart K, Christen M, Christen B, Letesson JJ, De Bolle X (2018). Transposon sequencing of *Brucella abortus* uncovers essential genes for growth in vitro and inside macrophages. Infect Immun.

[26] Aakre CD, Phung TN, Huang D, Laub MT (2013). A bacterial toxin inhibits DNA replication elongation through a direct interaction with the β sliding clamp. Mol Cell.

[27] Zhao BB, Li XH, Zeng YL, Lu YJ (2016). ClpP-deletion impairs the virulence of Legionella pneumophila and the optimal translocation of effector proteins. BMC Microbiol.

[28] Knudsen GM, Olsen JE, Aabo S, Barrow P, Rychlik I, Thomsen LE (2013). ClpP deletion causes attenuation of salmonella typhimurium virulence through mis-regulation of RpoS and indirect control of CsrA and the SPI genes. Microbiology.

[29] Frees D, Qazi SN, Hill PJ, Ingmer H (2003). Alternative roles of ClpX and ClpP in *Staphylococcus aureus* stress tolerance and virulence. Mol Microbiol.

[30] Frees D, Chastanet A, Qazi S, Sørensen K, Hill P, Msadek T, Ingmer H (2004). Clp ATPases are required for stress tolerance, intracellular replication and biofilm formation in *Staphylococcus aureus*. Mol Microbiol.

[31] Farrand AJ, Reniere ML, Ingmer H, Frees D, Skaar EP (2013). Regulation of host hemoglobin binding by the *Staphylococcus aureus* Clp proteolytic system. J Bacteriol.

[32] Robertson GT, Kovach ME, Allen CA, Ficht TA, Roop RM (2000). The *Brucella abortus* Lon functions as a generalized stress response protease and is required for wild-type virulence in BALB/c mice. Mol Microbiol.

[33] Park S, Choi YS, Park SH, Kim YR, Chu H, Hwang KJ, Park MY (2013). *Lon* mutant of *Brucella abortus* induces tumor necrosis factor-alpha in murine J774.A1 macrophage. Osong Public Health Res Perspect.

[34] Xiong X, Li B, Zhou Z, Gu G, Li M, Liu J, Jiao H (2021). The VirB system plays a crucial role in *Brucella* intracellular infection. Int J Mol Sci.

[35] Stahlhut SG, Alqarzaee AA, Jensen C, Fisker NS, Pereira AR, Pinho MG, Thomas VC, Frees D (2017). The ClpXP protease is dispensable for degradation of unfolded proteins in *Staphylococcus aureus*. Sci Rep.

[36] Frees D, Andersen JH, Hemmingsen L, Koskenniemi K, Bæk KT, Muhammed MK, Gudeta DD, Nyman TA, Sukura A, Varmanen P, Savijoki K (2012). New insights into *Staphylococcus aureus* stress tolerance and virulence regulation from an analysis of the role of the ClpP protease in the strains Newman, COL, and SA564. J Proteome Res.

[37] Roop RM (2012). Metal acquisition and virulence in *Brucella*. Anim Health Res Rev.

[38] Ferooz J, Lemaire J, Delory M, De Bolle X, Letesson JJ (2011). RpoE1, an extracytoplasmic function sigma factor, is a repressor of the flagellar system in *Brucella melitensis*. Microbiology.

[39] Delory M, Hallez R, Letesson JJ, De Bolle X (2006). An RpoH-like heat shock sigma factor is involved in stress response and virulence in *Brucella melitensis* 16M. J Bacteriol.

[40] Willett JW, Herrou J, Czyz DM, Cheng JX, Crosson S (2016). *Brucella abortus* ΔrpoE1 confers protective immunity against wild type challenge in a mouse model of brucellosis. Vaccine.

[41] Kirsch VC, Fetzer C, Sieber SA (2021). Global inventory of ClpP- and ClpX-regulated proteins in *Staphylococcus aureus*. J Proteome Res.

[42] Xie F, Zhang Y, Li G, Zhou L, Liu S, Wang C (2013). The ClpP protease is required for the stress tolerance and biofilm formation in *Actinobacillus pleuropneumoniae*. PLoS ONE.

[43] Paulley JT, Anderson ES, Roop RM (2007). *Brucella abortus* requires the heme transporter BhuA for maintenance of chronic infection in BALB/c mice. Infect Immun.

[44] Moreno-Cinos C, Goossens K, Salado IG, Van Der Veken P, De Winter H, Augustyns K (2019). ClpP Protease, a promising antimicrobial target. Int J Mol Sci.

