# Supplementary figures and images for: ClpP protease modulates bacterial growth, stress response, and bacterial virulence in Brucella abortus
Source: Vet Res. 2023 Aug 23;54:68. doi: 10.1186/s13567-023-01200-x (PMC10464072; doi:10.1186/s13567-023-01200-x)

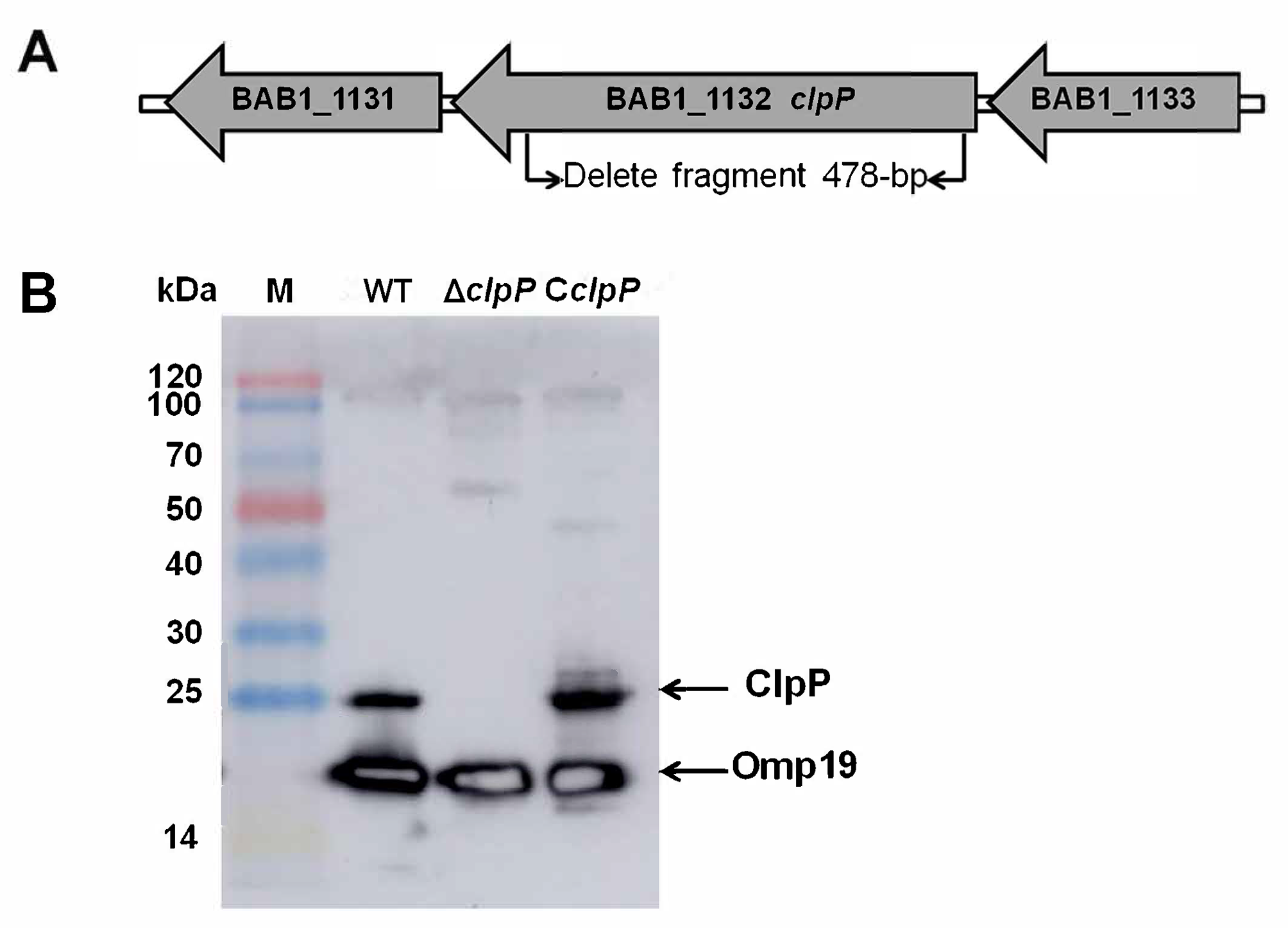

Supplement: Supplementary file 1 — Additional file 1: Characterization of the mutant strain ∆clpP. (A) Schematic of BAB1_1132 gene deletion. A 478-bp fragment was deleted from the BAB1_1132 coding sequence. (B) Westen blot assay of ClpP protein expression in different strain. Lane WT: protein sample of B.abortus 2308; Lane ∆clpP: protein sample of ∆clpP strain; Lane CclpP: protein sample of CclpP strain. [file 13567_2023_1200_MOESM1_ESM.tif]

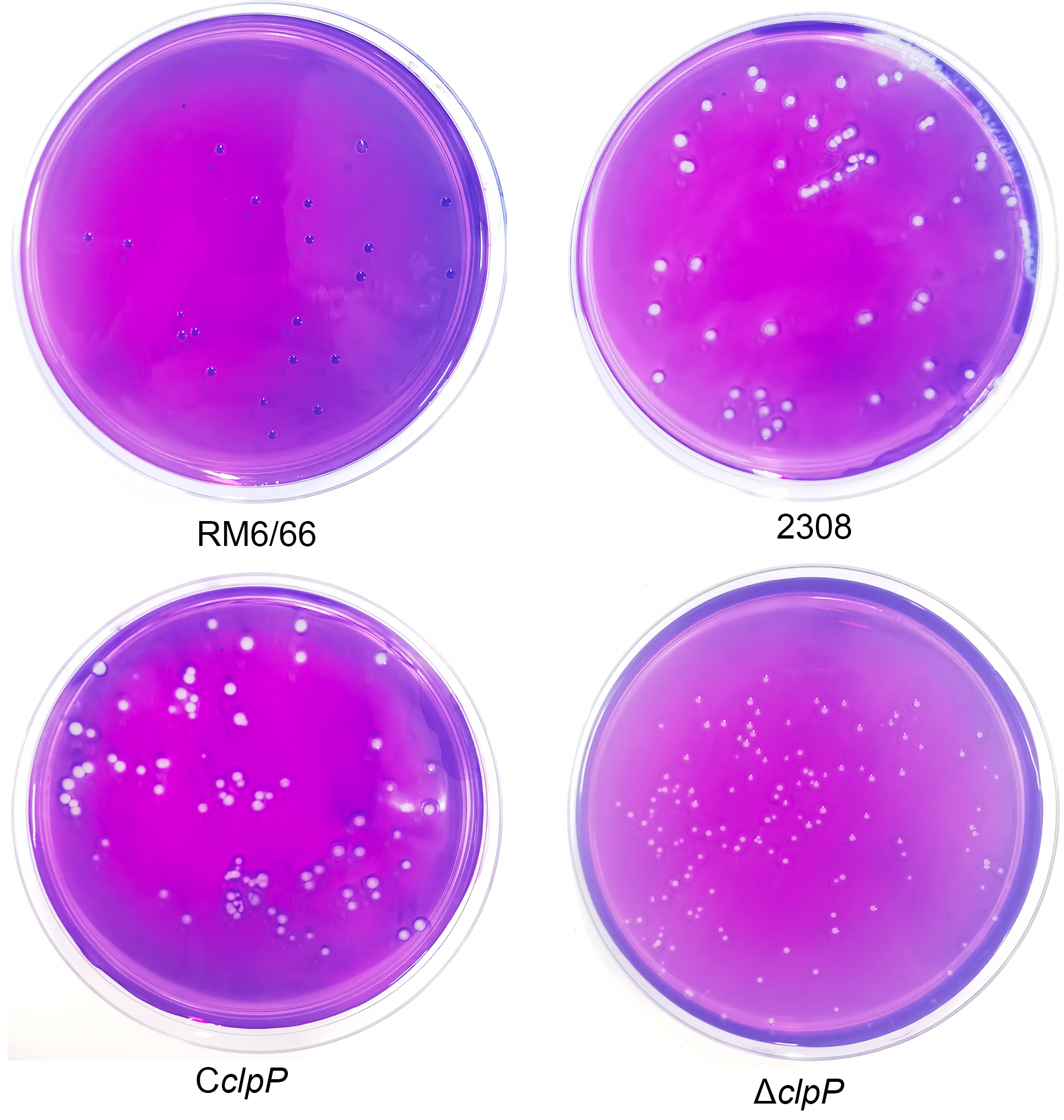

Supplement: Supplementary file 2 — Additional file 2: The crystal violet staining of Brucella strains. The brucella strains (2308, ∆clpP, CclpP, and RM6/66) were stained with 0.05% crystal violet. RM6/66 was a rough strain, which picked up the crystal violet dye. [file 13567_2023_1200_MOESM2_ESM.tif]
