# Supplementary material for: ClpP protease modulates bacterial growth, stress response, and bacterial virulence in Brucella abortus
Source: Vet Res. 2023 Aug 23;54:68. doi: 10.1186/s13567-023-01200-x (PMC10464072; doi:10.1186/s13567-023-01200-x)
Supplement: Supplementary file 3 — Additional file 3: Transcriptional level of 36 selected genes by RNA-seq and RT-qPCR. [file 13567_2023_1200_MOESM3_ESM.docx]

**Additional file 3 Transcriptional level of 36 selected genes by RNA-seq and RT-qPCR**

| Gene | Log_2_FC (△*clpP* vs 2308) | | Function |
| --- | --- | --- | --- |
|  | qPCR | RNA-seq |  |
| BAB2_0050 | 7.72 | 7.04 | Heat shock protein Hsp20 |
| BAB2_0773 | 5.34 | 4.17 | Secretion protein HlyD |
| BAB1_0446 | 4.45 | 3.33 | Heat shock protein DnaJ, N-terminal:Chaperone DnaJ, C-terminal:Heat shock protein DnaJ |
| BAB1_2129 | 4.74 | 3.29 | Heat shock protein Hsp70:Calcium-binding EF-hand |
| BAB1_1967 | 1.64 | 3.01 | Universal stress protein (Usp):Usp domain |
| BAB2_1082 | 5.90 | 2.80 | Ferric-uptake regulator |
| BAB1_0054 | 5.87 | 2.73 | TonB-dependent receptor protein:Mrp family:ATP/GTP-binding site motif A (P-loop):Protein of unknown function DUF59 |
| BAB2_0127 | 3.96 | 2.59 | flagellar basal-body rod protein FlgF |
| BAB2_0734 | 4.40 | 2.46 | Multiple resistance and pH regulation protein F |
| BAB1_1134 | 1.72 | 1.16 | Host factor Hfq |
| BAB1_1494 | -2.1 | -1.51 | immunoreactive 28 kDa outer membrane protein |
| BAB1_0322 | -0.43 | -2.66 | Secretion protein HlyD |
| BAB1_1451 | -1.17 | -2.93 | Cell cycle protein:Phosphopantetheine attachment site |
| BAB2_0709 | -2.45 | -3.06 | ATP/GTP-binding site motif A (P-loop):Cell divisionFtsK/SpoIIIE protein:Proline-rich extensin:AAA ATPase |
| BAB1_0722 | -4.22 | -4.86 | OmpA-like transmembrane domain |
| BAB2_0068 | -1.11 | -0.82 | SLT domain; LT_VirB1-like; VirB1-like subfamily |
| BAB2_0067 | -1.81 | -4.35 | type IV secretion system protein VirB2 KO: K03197 type IV secretion system protein VirB2 |
| BAB2_0066 | -0.50 | -0.50 | Type IV secretory pathway, VirB3-like |
| BAB2_0065 | 0.29 | -2.32 | Shikimate kinase:ATP/GTP-binding site motif A(P-loop):CagE, TrbE, VirB family component of type IV transporter system |
| BAB2_0064 | -1.04 | -2.57 | attachment mediating protein virB5 homolog KO: K03200 type IV secretion system protein VirB5 |
| BAB2_0063 | 0.57 | -0.50 | Beta and gamma crystallin:Pollen allergen PoapIX/Phl pVI, C-terminal:Sodium:dicarboxylate symporter:TrbL/VirB6 plasmid conju... |
| BAB2_0062 | -1.14 | -3.03 | type IV secretion system protein VirB7 KO:  K03202 type IV secretion system protein VirB7 |
| BAB2_0061 | -1.48 | -1.87 | VirB8 |
| BAB2_0060 | -2.41 | -1.48 | Type IV secretion system CagX conjugation  protein |
| BAB2_0059 | 0.07 | -2.14 | Glutelin:Proline-rich region:Bacterial conjugation TrbI-like protein |
| BAB2_0058 | 0.06 | -1.48 | Bacterial type II secretion system protein E:ATP/GTP-binding site motif A (P-loop):Sigma-54 factor interaction domain |
| BAB2_0012 | 1.04 | -0.19 | Short-chain dehydrogenase/reductase SDR:Blood group Rhesus C/E and D polypeptide:Glucose/ribitol dehydrogenase |
| BAB2_0013 | 0.40 | -0.30 | Isochorismatase hydrolase family:Isochorismatase:Phosphopantetheine attachment site:Phosphopantetheine-binding domain |
| BAB2_0014 | 0.57 | -0.06 | Eubacterial/plasma membrane H+-transporting two-sector ATPase, C subunit:AMP-dependent synthetase and ligase |
| BAB2_0233 | -0.51 | -0.26 | TonB-dependent receptor protein |
| BAB2_0519 | 0.47 | -0.41 | Bacterial periplasmic spermidine/putrescine-binding protein:Gliadin, alpha/beta:Bacterial extracellular solute-binding protei... |
| BAB2_0539 | 1.26 | 0.18 | Bacterial extracellular solute-binding protein, family 1 |
| BAB2_0564 | 0.10 | 0.35 | Periplasmic binding protein |
| BAB2_0675 | 0.61 | -0.67 | Ferritin:Bacterioferritin |
| BAB2_1150 | -0.10 | 1.80 | TonB-dependent receptor protein:Pollen allergen Poa pIX/Phl pVI, C-terminal |
| BAB1_1672 | 1.24 | 1.02 | Sigma factor, ECF subfamily:Sigma-70 region 2:Sigma-70 region 4 |
