# Supplementary material for: ClpP protease modulates bacterial growth, stress response, and bacterial virulence in Brucella abortus
Source: Vet Res. 2023 Aug 23;54:68. doi: 10.1186/s13567-023-01200-x (PMC10464072; doi:10.1186/s13567-023-01200-x)
Supplement: Supplementary file 4 — Additional file 4: Primers used in this study. [file 13567_2023_1200_MOESM4_ESM.doc]

**Additional file 4 Primers used in this study**

| Primers | Sequences (5’to 3’) | Used for |
| --- | --- | --- |
| DclpP-F1 | GGggtaccGAAACACGGATAGCAGGTG | Construct the *clpP* mutant |
| DclpP-R1 | TTATTTCGATTCGTCTGCGggatccACGGGTCCATTGACGAA |
| DclpP-F2 | TTCGTCAATGGACCCGTggatccCGCAGACGAATCGAAATAA |
| DclpP-R2 | GGgagctcAACCGCAACCGACAGAA |
| Kan-F | ggatccCACGTCTTGAGCGATTGTGTAG |  |
| Kan-R | ggatccCGGAAAACGATTCCGAAGCCC |  |
| CclpP-F | CGgaattcGCGGATATTTCGCGCCAC | Construct the complemented strain |
| CclpP-R | GGggtaccCAAAGATTCGGCTCATTCC |
| rClpP-For | CCgagctcATGAGAGATCCGATCGAAACCGTCA | Construct the ClpP recombinant expression plasmids |
| rClpP-Rev | GGaagcttTTTCGATTCGTCTGCGCTTACATCG |
| 16S rRNAf | TACCAGCCCTTGACATCC | RT-qPCR |
| 16S rRNAr | TCATCCCCACCTTCCTCT |
| BAB2_0050-F | AAAACGCCTATCGCATCA |
| BAB2_0050-R | TTCATCTGTTCTGGAATCTCG |
| BAB2_0773-F | TCATTCCGTTCGTTTCATC |
| BAB2_0773-R | TCCTGGCATCCGTATCTT |
| BAB1_0446-F | CATTGACGATGCGGTTCT |
| BAB1_0446-R | TGATGCGGACATGGACATA |
| BAB1_2129-F | GAACGCAACACCACTATCC |
| BAB1_2129-R | GGCTGCCATTTCACGCT |
| BAB1_1967-F | CGGCGTGGTGTTTGTTT |
| BAB1_1967-R | GCAGTCCTTCGCTTTCG |
| BAB2_1082-F | CAGTTACGGGACGATGGC |
| BAB2_1082-R | ACGAAGGCGTTGAGGCT |
| BAB2_0127-F | GACAAGCAGGCGAAAATG |
| BAB2_0127-R | AGAAACGATGCGATGGAG |
| BAB2_0734-F | GCTTGCCGCTCAGGTCA |
| BAB2_0734-R | GCATCGGTCGCCAGAATA |
| BAB1_1134-F | TCGCAAAATCTACAAGACCTC |
| BAB1_1134-R | CTTCCTCGCCTTCAAACA |
| BAB1_0322-F | TACGGAAGCAGCGGAAAT |
| BAB1_0322-R | CAAGCGAGGACGAGGTG |
| BAB1_1451-F | GGCTTTTGTTGTCGTCTGC |
| BAB1_1451-R | CAAGGTCGGGCTGGAGTA |
| BAB1_0054-F | TGGAGCGGCTGAAAACT |
| BAB1_0054-R | GAACCGTGATGGAGAAGAAG |
| BAB2_0709-F | TGCGACTACAACCACAACG |
| BAB2_0709-R | TGCGACGACTGCTACCG |
| BAB1_0722-F | CGCCATCCAGGAACAGC |
| BAB1_0722-R | CCAGCCATAGCCAAGGTAA |
| BAB1_1494-F | AACAACCTGAAAGAGCCTACC |  |
| BAB1_1494-R | CCTGATTAACACCGAGCGT |
| BAB2_0068-F | TTCGCGCCGCATTCTCCTGT |  |
| BAB2_0068-R | GCGTTTTCCGGATCATGGGC |  |
| BAB2_0067-F | CAGCACCGGCACTACATCCA |  |
| BAB2_0067-R | GCTAAGCGGCGTATCGATCA |  |
| BAB2_0066-F | AGGCAATCGGGCTATAGGCG |  |
| BAB2_0066-R | GGCAGATCACGCAGACTGAC |  |
| BAB2_0065-F | AAAACACCACTTCGCGAGCC |  |
| BAB2_0065-R | TGACCACAACGGACGAAAGC |  |
| BAB2_0064-F | GCTTTCGATCCGGCCTTGCA |  |
| BAB2_0064-R | AGTGGTGAAGCCGGTCCAGG |  |
| BAB2_0063-F | ACTGTCATTCCTGGTAGCCG |  |
| BAB2_0063-R | ATGTGACGATGGCCCCGAAC |  |
| BAB2_0062-F | CCTTGCTTTTGTCGCCACGG |  |
| BAB2_0062-R | CCGCGGTTTGCCATCGAGAA |  |
| BAB2_0061-F | GCCTTATCGCCCTGGAACTG |  |
| BAB2_0061-R | CGACTGGTACACGCTGCAAA |  |
| BAB2_0060-F | ACATGCGCTGATCTGTCGTG |  |
| BAB2_0060-R | CCGGGCGAGAATATTGAGGA |  |
| BAB2_0059-F | CTTCGCGAGCAGGAAATCAC |  |
| BAB2_0059-R | GATACGAACGCTCAAACGGC |  |
| BAB2_0058-F | AGGTGGACGTGGTTCGGATG |  |
| BAB2_0058-R | GACGGGATCAGGCAAGACCA |  |
| BAB2_0012-F | CATCGAAAGCAGCATTGGCA |  |
| BAB2_0012-R | CCGGAAGGATCGTTCAGCAT |  |
| BAB2_0013-F | CCTTGCGCTGAAATATGTCG |  |
| BAB2_0013-R | TTTTGCAGCCAGTCGCGGGT |  |
| BAB2_0014-F | AACCGCTCAACTGCTTCTCC |  |
| BAB2_0014-R | CCACCTGCACGAGTTTCAGC |  |
| BAB2_0233-F | ACCTATGATGAGCCTCTGCC |  |
| BAB2_0233-R | GTCCTTGCCCACCACATTGC |  |
| BAB2_0519-F | CATCAAGGGCGCGAAGAACC |  |
| BAB2_0519-R | TGGAACCTCGGCAGACTTGT |  |
| BAB2_0539-F | ACTCACCCGCCGATATCCTG |  |
| BAB2_0539-R | ATACCAATCGCCGTCCGCAT |  |
| BAB2_0564-F | TGCCGCGTTGAAGAAGATCG |  |
| BAB2_0564-R | CCTTGGCTTCCGCTTCCTTG |  |
| BAB2_0675-F | CGAGGCACTGTTTCTTGAGC |  |
| BAB2_0675-R | GTGATGCATTTCCTCGATGG |  |
| BAB2_1150-F | TCTGCCGAATTATGACAGCC |  |
| BAB2_1150-R | GCGCCAATGGTCGTGTAGTT |  |
| BAB1_1672-F | TCAGGATTTTCGCGCTGCCC |  |
| BAB1_1672-R | AAATCTCGGCCGCTTCCTCA |  |
